# Transfer Entropy Causal Networks for Interconnectedness Analysis of Global Banking and Green Markets: A CEEMDAN-SE-KM Approach

**DOI:** 10.3390/e28070814

**Published:** 2026-07-17

**Authors:** Qiuyang Xue, Xiu Jin, Jinming Yu, Yueli Liu

**Affiliations:** School of Business Administration, Northeastern University, Shenyang 110169, China; xueqy@mails.neu.edu.cn (Q.X.); 15204677362@163.com (J.Y.); 2110433@stu.neu.edu.cn (Y.L.)

**Keywords:** transfer entropy, causal networks, CEEMDAN-SE-KM, green markets, banking sectors

## Abstract

In light of growing concerns about sustainable development and green innovation, the green market has progressively taken center stage in the financial markets. From the nonlinear information transmission angle, we look into the interconnectedness between the global banking sectors and the green markets using transfer entropy causal networks, containing the Dow Jones Green Bond Index (SPGB), Dow Jones Sustainability Index (DJSI), The S&P Global Clean Energy Index (SPCL), and MSCI World ESG Leaders Index (ESGL). We observe significant bidirectional causal relationships between two markets. The banking industries of developed nations and emerging economies like South Korea, Indonesia, and India are the most important, while four green markets are vital. Furthermore, using the CEEMDAN-SE-KM approach, this study also investigates the two markets’ heterogeneous performance at various time scales. The causal relationships between two markets exhibit heterogeneity at time scales, and that is most noticeable at the short-term scale. Additionally, after the COVID-19 pandemic and the conflict between Russia and Ukraine, there is an increase in the causal relationships between the two markets and a higher efficiency of information transmission. These results help regulatory bodies and green market players have a more thorough understanding of and dynamic regulation of the green market.

## 1. Introduction

Environmental degradation and climate change have emerged as some of the most significant challenges facing humanity. In response, national governments have implemented effective measures to reduce carbon emissions and fuel consumption, aiming to prevent potential environmental disasters [1]. Concurrently, the promotion of green economic growth has become a collective global objective in the pursuit of sustainable development. A major focus of recent United Nations climate change conferences, such as COP26 in Glasgow and COP27 in Sharm El Sheikh, has been green finance. Financial institutions worldwide have developed investment opportunities in green markets, including green bonds, clean energy markets, and sustainable and ESG markets [2,3,4,5]. Green finance, as an effective means of funding climate-oriented projects to achieve a low-carbon economy, has been extensively documented [6,7,8]. The interconnectedness between green investments and other financial markets has garnered significant research attention in recent years, driven by the growing number of market participants in green projects and a marked rise in allocations to green investments [9,10,11,12].

The global banking sector plays a pivotal role in facilitating financial support for green innovation and addressing the challenges posed by climate change [13]. National banking industries constitute a substantial portion of a country’s Gross Domestic Product (GDP) and investment, with the global banking sector consistently delivering financial solutions to multinational corporations [14]. In the aftermath of the global financial crisis (GFC), the banking sector has functioned as a conduit for economic shocks, contributing to global financial imbalances and recessionary trends. Consequently, the significance of the banking sector within financial markets is unequivocal. Recent scholarly investigations have predominantly concentrated on the intricate interconnections between the global banking sector and other markets [15,16,17,18,19,20].

Despite the evident interconnectedness between banking sectors and green markets, this relationship remains insufficiently explored. The theoretical rationale for investigating the transfer entropy relationship between these sectors can be elucidated through various economic transmission channels. Firstly, the financing channel posits that banks serve as a primary source of external capital for green initiatives, offering green loans, project financing for clean energy, and underwriting services for green bonds. Consequently, fluctuations in banks’ funding costs, lending capacity, and risk appetite are transmitted to green assets via the cost and availability of capital, while the expansion of green markets simultaneously generates new lending opportunities and fee income for banks [13]. Secondly, the balance-sheet channel suggests that banks, by holding green bonds and equities of clean-energy and ESG firms, and by lending to both green and carbon-intensive borrowers, experience feedback effects from price changes in green markets, which impact bank asset values and credit quality. Additionally, shocks to banks can propagate to the green assets they finance and hold [21]. The intricate interrelation between these two markets underscores the necessity for further scholarly inquiry.

The studies in the available reviews essentially employ traditional econometric methods to study the interdependent relationship between green markets and traditional markets [11,22,23,24]. Nonetheless, information transmission in financial markets inherently exhibits nonlinear characteristics [25,26]. Transfer entropy (TE), a data-driven approach to information dynamics, has gained considerable traction in the field of finance in recent years [27,28]. As a generalization of Granger causality, transfer entropy simplifies to the latter in the context of linear Gaussian processes. It is capable of capturing any statistical feature, including asymmetric and nonlinear information transfer within systems, which cannot be detected by the Vector Autoregression (VAR) method [29,30]. Recent financial studies have employed TE to investigate information transmission and causal relationships across various markets [31,32]. In this paper, we utilize the transfer entropy method to construct a causal network between global banking sectors and green markets, and we conduct a detailed analysis of the interconnected network’s structure.

Furthermore, frequency-domain analysis can aid market participants in discerning the heterogeneity and intricate dynamics of the relationships between global banking sectors and green markets across various time scales [33]. The expectations, sentiments, and risk preferences of market participants are subject to temporal evolution [34]. The CEEMDAN method has been utilized for the adaptive decomposition of financial time series [35,36,37]. Notably, several studies have conducted comprehensive comparisons between EMD-based approaches and traditional techniques, demonstrating that EMD-based methods surpass Fourier and wavelet transforms in several respects [38,39]. A significant advantage of CEEMDAN in the context of financial time series is its capacity to mitigate the persistence of noise, which is often attributed to the behavior of irrational investors [40]. Its superiority over traditional EMD lies in its ability to eliminate modal aliasing [41]. The CEEMDAN method achieves its objective by conducting multiple decompositions with varying noise realizations and subsequently averaging the outcomes. This ensemble averaging process effectively mitigates the influence of added noise while preserving the stable components of the original time series. This study utilizes the CEEMDAN technique for time series decomposition, calculates the complexity of the decomposed series using sample entropy (SE) [42,43], and applies the k-means (KM) algorithm for reorganizing the series based on varying complexity levels [44]. Specifically, we implemented the CEEMDAN-SE-KM approach to decompose and reorganize the time series into short-, medium-, and long-term components, facilitating a multiscale analysis of the causal relationships between global banking sectors and green markets.

Currently, the world is confronting emerging crises. The COVID-19 pandemic represents a global public health emergency, while the Russia–Ukraine conflict constitutes a war-related crisis, with significant instability observed among Russia’s major trading partners following the conflict’s onset. The repercussions of these two black swan events have resulted in an escalation of the magnitude of international economic shocks. The shocks from these two black swan events have led to a surge in the magnitude of interconnectedness among financial assets, exerting a serious impact on the global financial market [45,46,47,48,49,50]. A number of studies have provided evidence of increased connectivity between green indices following the onset of these two crisis events. This is also the case for the banking sector [19,51,52,53,54]. Therefore, the impact of the outbreak of crisis events on the interconnectivity between financial markets should be taken seriously. This paper explores the changes in the causal relationships between the two markets before and after the outbreak of the COVID-19 pandemic and the Russia–Ukraine conflict.

From the perspective of nonlinear information transmission, this study introduces the transfer entropy method to construct a causal network and compute topological indicators, thereby analyzing the interconnected relationships between global banking sectors and green markets. The research encompasses sixteen developed and emerging countries, along with four green investment indices. Additionally, the CEEMDAN-SE-KM approach is utilized to explore the multiscale characteristics of the causal relationship between these two markets. Furthermore, the study examines the evolution of associations and information transmission efficiency between global banking sectors and green markets in the context of the COVID-19 pandemic and the Russia–Ukraine conflict.

The findings of the study indicate that the relationship between global banking sectors and green markets is intricate. There exists a substantial nonlinear transmission of information between these two markets. All four green investment indices demonstrate a significant association with the banking sectors. Among these indices, the S&P Dow Jones Green Bond Index (SPGB), the Dow Jones Sustainability Index (DJSI), and the MSCI World ESG Leaders Index (ESGL) are pivotal within the transfer entropy causal network. Consequently, it is imperative for market regulators to enhance their oversight of green markets to mitigate the risk of these markets transmitting shocks to others. Notably, the critical banking sectors are predominantly located in developed countries, with India, Indonesia, and South Korea representing key emerging markets. As both transmitters and receivers of information, the interconnectivity and complexity of these sectors with other markets warrant careful consideration. Moreover, the causal relationships between banking sectors and green markets exhibit heterogeneity across various time scales, necessitating that market participants develop a comprehensive understanding of green investments and regulatory frameworks across these different temporal dimensions. In conclusion, the onset of the COVID-19 pandemic and the Russia–Ukraine conflict have led to an increase in causality and enhanced information transmission efficiency between the two markets. Furthermore, there is a noticeable increase in the information flow between the banking sectors and the green markets since the COVID-19 pandemic outbreak. Regulatory authorities should adopt different strategies in response to crises of varying natures.

This study makes contributions to several strands of literature. Firstly, it encompasses an analysis of 16 global banking sectors and four major global green benchmark indices, utilizing a directed transfer entropy causal network to elucidate the global bidirectional nonlinear linkages between banking and green markets. Secondly, the study integrates Complete Ensemble Empirical Mode Decomposition with Adaptive Noise (CEEMDAN), sample entropy (SE), and k-means clustering to develop a fully data-driven multiscale decomposition framework. This approach circumvents subjective frequency segmentation and enhances the accuracy of multiscale information transmission identification. Third, this study unifies COVID-19 (global public health crisis) and the Russia–Ukraine conflict (geopolitical war crisis) within the same network framework, comparing their differentiated impacts on cross-market causality and information transmission efficiency. Several entropy-based studies have examined financial markets during crisis periods. Sheraz and Nasir [55] compare information-theoretic measures for modeling stock market volatility. Drzazga-Szczesniak et al. [56] use Shannon entropy to study the effect of the Russia–Ukraine war on the Polish stock market. Drzazga-Szczesniak et al. [57] further identify extreme-event signatures through a cumulative entropic spectrum. These studies show that entropy is useful for detecting volatility changes and event-related market patterns. Rather than using entropy mainly as a volatility or event-detection measure for a single market or index, we use transfer entropy to identify nonlinear and asymmetric information flows between global banking sectors and green markets.

The remainder of the paper is structured as follows. Section 2 shows the method. Section 3 describes the data. Section 4 discusses the results. Section 5 concludes.

## 2. Methods

### 2.1. CEEMDAN-SE-KM

#### 2.1.1. CEEMDAN

Financial time series data are highly non-stationary and nonlinear. We employ the Complete Ensemble Empirical Mode Decomposition with Adaptive Noise (CEEMDAN) to process the data of the banking sector and green investment indices. This facilitates the understanding and analysis of the dynamic behavior of financial markets, which exhibit characteristics at multiple time scales.

The CEEMDAN method overcomes issues such as endpoint effects and mode mixing that may arise when dealing with complex signals using the Empirical Mode Decomposition (EMD). Additionally, this method introduces the capability to handle both positive and negative Gaussian white noise, enhancing the Ensemble Empirical Mode Decomposition (EEMD) algorithm and thereby reducing the error in signal reconstruction. The CEEMDAN algorithm can be divided into the following steps:

(1) Add Gaussian white noise to the original series $R_{i,t}$ *N* times, resulting in *N* pre-processed series:

(1) $$s_{n}(t)=R_{i,t}+\varepsilon_{0}\omega_{k}(t)$$

where $\varepsilon_{0}$ stands for the weight coefficient of the Gaussian white noise and $\omega_{k}(t)$ denotes the Gaussian white noise at the *k*-th processing stage.

(2) Empirical Mode Decomposition (EMD) is conducted on all pre-processed time series $s_{n}\left(t\right)$ to extract the first intrinsic mode function (IMF) component, denoted as ${IMF}_{1}^{k}(t)$. Next, ${\bar{IMF}}_{1}(t)$, the average of these IMFs, is taken as the first IMF component in the CEEMDAN. Along with this, $r_{1}(t)$, the first residual sequence is derived. The ${\bar{IMF}}_{1}(t)$ and $r_{1}(t)$ were calculated by the following Equations (2) and (3):

(2) $${\bar{IMF}}_{1}\left(t\right)=\frac{1}{N}\sum_{1}^{N}{IMF}_{1}^{k}(t)$$

(3) $$r_{1}\left(t\right)=R_{i,t}-{\bar{IMF}}_{1}\left(t\right)$$

(3) Introduce Gaussian white noise into $r_{1}\left(t\right)$ to create *N* new series, which are expressed as $r_{1}\left(t\right)+\varepsilon_{1}E_{1}(\omega_{k}(t))$. Apply EDM on these *N* series and their average is calculated as the second IMF, denoted as ${\bar{IMF}}_{2}(t)$ with the Equation (4):

(4) $${\bar{IMF}}_{2}\left(t\right)=\frac{1}{N}\sum_{1}^{N}E_{1}(r_{1}\left(t\right)+\varepsilon_{1}E_{1}(\omega_{k}(t))$$

Next, the residual $r_{2}\left(t\right)$ is obtained. For m=2,…,M, the residual at the *m*-th stage is calculated as below:

(5) $$r_{m}\left(t\right)=r_{m-1}\left(t\right)-{\bar{IMF}}_{m}\left(t\right)$$

(4) After *N* times EDM decomposition of $r_{m}\left(t\right)+\varepsilon_{m}E_{m}\left(\omega_{k}\left(t\right)\right)$, results in the (*m* + 1)-th IMFs after CEEMDAN modal decomposition, which is shown as follows:

(6) $${\bar{IMF}}_{m+1}\left(t\right)=\frac{1}{N}\sum_{1}^{N}E_{1}(r_{m}\left(t\right)+\varepsilon_{m}E_{m}(\omega_{k}(t))$$

(5) Repeat step (4) until $r_{m}\left(t\right)$ is no longer decomposed. The final residue is obtained:

(7) $$r_{M}\left(t\right)=R_{i,t}-\sum_{1}^{N}{\bar{IMF}}_{m}\left(t\right)$$

and the exact decomposition of the original series $R_{i,t}$ is obtained:

(8) $$R_{i,t}=r_{M}\left(t\right)+\sum_{1}^{N}{\bar{IMF}}_{m}\left(t\right)$$

#### 2.1.2. Simple Entropy (SE)

Sample entropy [58] is a measure used to quantify the complexity of a signal. This metric can intuitively reflect the complexity of the time series; the lower the SE value, the higher the self-similarity, indicating that the time series is less complex. The process for calculating SE is as follows:

(9) $$SamEn\left(m,r,L\right)=\underset{L\to \infty }{\lim}\left\{-ln[A^{m}(r)/B^{m}(r)]\right\}$$

where *L* denotes the length of the time series, *r* stands for the similarity tolerance, *m* is the embedding dimension; $A^{m}(r)$ and $B^{m}\left(r\right)$ represent the probabilities of two time series matching *m* + 1 and *m* points at a threshold *r*. The computation of SE does not rely on the length of the time series. Thus, SE is more appropriate for determining the complexity of financial time series than approximate entropy.

#### 2.1.3. K-means (KM)

By utilizing the CEEMDAN method to decompose the return data and calculating the complexity using SE, the next step is to cluster the data based on their varying complexities. We employ the classical k-means (KM) clustering method to classify return data with different complexities.

The KM algorithm works on the following principle: given a set of samples *S* and the number of clusters *k*, the algorithm divides *S* into *k* clusters based on the similarity of data complexities. It then updates the cluster centers iteratively to adjust the classification until the overall difference function converges, automatically selecting the optimal result among all clusters.

In this study, we chose *k* = 3, which corresponds to capturing the information at three time scales: short-, medium-, and long-term. By employing the CEEMDAN-SE-KM method of decomposition and reconstruction, we can more clearly delineate the interconnectedness between banking sectors and green assets at different time scales.

### 2.2. Transfer Entropy Causal Network Construction

In information theory, Shannon entropy H(X) states the average uncertainty of a sample of a discrete random variable, equivalent to the average amount of information required to predict the value of a discrete random variable. The magnitude of H(X) is uniquely determined by the probability mass function p(x) of *X*, which can be calculated as $H\left(X\right)=-\sum_{x\in X}p\left(x\right){log}_{2}p\left(x\right)$. For two discrete random variables (X,Y), the joint entropy is simply: $H\left(X,Y\right)=-\sum_{x}\sum_{y}p\left(x,y\right){log}_{2}p\left(x,y\right)$; conditional entropy represents the information content of *Y* given that *X* occurs: $H\left(Y|X\right)=-\sum_{x}\sum_{y}p\left(x,y\right){log}_{2}p\left(y|x\right)$. Transfer entropy (TE) measures the asymmetric information transfer between subsystems in the complex system, the information transfer from process *Y* to *X* is shown as follows:

(10) $${TE}_{Y\to X}\left(k,l\right)=H_{X}\left(k\right)-H_{XY}\left(k,l\right)=\sum_{x_{t+1,x_{t}^{\left(k\right)},y_{t}^{\left(l\right)}}}p\left(x_{t+1},x_{t}^{\left(k\right)},y_{t}^{\left(l\right)}\right){log}_{2}\frac{p\left(x_{t+1}|x_{t}^{\left(k\right)},y_{t}^{\left(l\right)}\right)}{{log}_{2}p\left(x_{t+1}|x_{t}^{\left(k\right)}\right)}$$

where $H_{X}\left(k\right)=-\sum_{x_{t+1,x_{t}^{\left(k\right)},y_{t}^{\left(l\right)}}}p\left(x_{t+1},x_{t}^{\left(k\right)},y_{t}^{\left(l\right)}\right){log}_{2}p\left(x_{t+1}|x_{t}^{\left(k\right)}\right)$, $H_{X}\left(k\right)$ is the conditional entropy when *k*th-order time-lag series $x_{t}^{\left(k\right)}=(x_{t},\ldots x_{t-k+1})$ of the random process *X* is known; $H_{XY}\left(k,l\right)=-\sum_{x_{t+1,x_{t}^{\left(k\right)},y_{t}^{\left(l\right)}}}p\left(x_{t+1},x_{t}^{\left(k\right)},y_{t}^{\left(l\right)}\right){log}_{2}p\left(x_{t+1}|x_{t}^{\left(k\right)},y_{t}^{\left(l\right)}\right)$ is the conditional entropy when both *k*th-order time-lag series of the random process *X* and lth-order time-lag series of the random process *Y* are known.

This paper uses the toolbox IDTxl to calculate the transfer entrpy [59], which enables us to construct the probability distribution functions via a kernel density estimator (KDE). Let $u_{j}$, $u_{j}$, …$u_{j}$ be a sample of U ∈ $R^{d}$, *h* is the bandwidth and the probability density function value $\hat{p}\left(u_{j}\right)$ could be estimated by KDE with a kernel function *K* (·) as

(11) $$\hat{p}\left(u_{j}\right)=\frac{1}{Nh^{d}}\sum_{i=1}^{n}K\left(\frac{u_{j}-u_{i}}{h}\right)$$

We estimate the probability densities in Equation (10) with a kernel density estimator (KDE) rather than a histogram, because the KDE does not require the data to be grouped into discrete bins and therefore avoids the loss of information that the choice of bin width would introduce [60]. Following [60], we use a Gaussian kernel, which is well-suited to the continuous and heavy-tailed nature of financial returns. The bandwidth *h* controls the degree of smoothing: a value that is too small gives a noisy estimate, whereas a value that is too large over-smooths the density and hides the dependence between series. To avoid manual tuning, we set *h* with Silverman’s rule of thumb, $h={\left(4\sigma^{5}/3n\right)}^{1/5}$, where σ is the standard deviation of the series and n is its length. This data-driven choice balances bias and variance and keeps the density estimation consistent across all series.

The history lengths k in Equation (10) set how many past states of the target series are conditioned on. Because the data are sampled at the daily frequency, at which financial information is transmitted quickly, we restrict the analysis to short lags of k = 1, 2, and 3 days [61]. When a causal link between two series is significant at more than one lag, we report it at the largest significant lag, so that the network reflects the longest horizon over which information is still transmitted, and the same link is not counted more than once.

The finite-sample estimator of TE is biased, partly because series with higher entropy, which in finance corresponds to higher volatility, mechanically appear to transfer more information. To address this bias, we assess the statistical significance of each estimate against a surrogate distribution, using the procedure implemented in the IDTxl toolbox [59] and applied to multivariate financial networks by [61]. For each ordered pair of series, the source series is randomly shuffled *n* = 200 times; shuffling removes the temporal coupling between the two series while preserving their marginal distributions, so the transfer entropy computed on the shuffled data represents the value expected under the null hypothesis of no information transfer. The observed transfer entropy is then compared with this null distribution, and a directed link is retained, with *M_ij_* = 1 only when the observed value exceeds the null distribution at the 0.05 significance level; otherwise, *M_ij_* = 0. The use of 200 surrogates and a 5% significance level is consistent with previous transfer entropy studies [59,61] and balances the resolution of the test against its computational cost. The resulting adjacency matrix *M* defines the directed and unweighted causal network. A binary adjacency matrix *M* will be obtained. Then, a directed unweighted TE causal network will be constructed based on the adjacency matrix *M*.

### 2.3. Network Topological Features

#### 2.3.1. Total-Out-To-Other

Total-Out-To-Other (TOTO) examines the magnitude of the interactions between the banking sectors and the green markets [62]. The expression is as follows:

(12) $$TOTO\left(j\right)=\sum_{i=1}^{N-N_{m}}E_{j\to i},\;iV\backslash \left\{j|jm\right\}$$

where $N_{m}$ indicates the number of nodes belonging to the TE causal network. “TOTO” examines the total number of TE causal relations formed by all nodes *j* belonging to banking sectors to all nodes *i* belonging to the green markets, and vice versa.

#### 2.3.2. Network Density

We use network density (NE) to characterize the level of information transmission efficiency between banking sectors and green markets in TE causal networks. It is calculated as Equation (13):

(13) $$ND=\frac{L}{N(N-1)}$$

where *L* denotes the actual number of directed edges in the TE networks, *N* is the number of nodes and *N*(*N* − 1) indicates the maximum possible number of directed edges. The range of network density is [0, 1]. The higher the efficiency of information transmission, the higher the network density.

## 3. Data

The study explores the interconnectedness between global banking sectors and green markets. The stock indexes of global banking sectors considered in this paper cover sixteen countries. The developed countries include the United States (US), the United Kingdom (UK), France (FR), Germany (DE), Japan (JP), Italy (IT), Canada (CA), and Australia (AU). Meanwhile, the developing countries selected are Argentina (AR), Brazil (BR), Turkey (TR), China (CN), South Korea (KR), Indonesia (ID), India (IN), and South Africa (ZA). These countries encompass participants from all continents and hold significant influence in international trade and the economic sphere. Moreover, as the emphasis on ESG investments grows, the role of the banking sectors in these countries in promoting sustainable investments and green finance is drawing more and more attention.

On the other hand, we select four indices to represent green investment [1], including: (i) The S&P Dow Jones Green Bond Index (SPGB), a market-value-weighted index that encompasses bonds from any nation and currency, provided they are certified as green by the Climate Bonds Initiative. This comprehensive index includes a range of debt instruments such as sovereign bonds, government-related, corporate, and securitized issues. (ii) The Dow Jones Sustainability Index (DJSI) is recognized as the world’s first benchmark for sustainable investments. It exclusively comprises companies that achieve the highest scores in sustainability evaluations, making the DJSI a popular benchmark for investors prioritizing sustainability in their portfolio construction. (iii) The S&P Global Clean Energy Index (SPCL) tracks the performance of clean energy companies operating worldwide, including both developed and emerging markets. These companies are primarily engaged in activities related to clean energy equipment and technology. (iv) The MSCI Global ESG Leaders Index (ESGL), created by Morgan Stanley Capital International (MSCI), is designed to reflect the performance of companies that have higher ratings in environmental, social, and corporate governance (ESG) performance compared to their counterparts.

Data on equity and green markets are collected from Thomson Reuters DataStream, with DataStream codes of the data series webpage https://www.lseg.com/en/data-analytics/datastream-and-macroeconomics, accessed on 14 July 2026. All indices are denominated in U.S. dollars. The returns are calculated by $R_{i,t}=ln(P_{i,t}/P_{i,t-1})$, where $P_{i,t}$ denotes the price of the index *i* on day *t*. The dataset of this study spans from 1 January 2018, to 31 March 2024, comprising a total of 1627 observations. Following [61], we employ third-order spline interpolation to fill the time series gaps. These gaps mainly reflect non-overlapping public holidays across the sixteen national markets and are short and dispersed rather than systematic, so third-order spline interpolation preserves trend continuity without introducing artificial jumps in the return series.

Table 1 reports the descriptive statistics of the daily returns of the sixteen banking sectors and four green indices. The mean returns are close to zero, while volatility differs across markets. SPGB has the lowest standard deviation, whereas Argentina and Turkey show the highest volatility, indicating greater instability in emerging banking markets. Most return series are negatively skewed, and all series have kurtosis above three, suggesting fat-tailed distributions. The Jarque–Bera test rejects normality for all series at the 1% level, and the ADF test confirms that all return series are stationary. These results indicate non-normal and heavy-tailed return behavior, supporting the use of transfer entropy to capture nonlinear and asymmetric information transmission between banking sectors and green markets.

This research further examines two unprecedented crisis periods of distinct natures: the COVID-19 pandemic and the Russia–Ukraine conflict. The date 31 December 2019 is used to split the COVID-19 pandemic period into pre-COVID-19 (1 January 2019 to 30 December 2019) and during-COVID-19 (1 January 2020 to 31 December 2020). Also, the Russia–Ukraine conflict was divided into two periods: pre-Conflict (1 January 2021 to 23 February 2022) and during-Conflict periods (24 February 2022 to 31 December 2022) based on the Russia–Ukraine conflict outbreak date of 24 February 2022. For the COVID-19 pandemic, 31 December 2019 is treated as the key public information date, because this was when pneumonia cases in Wuhan were first reported to the WHO. Therefore, observations before this date are classified as the pre-COVID-19 period, while the year 2020 is used to represent the main pandemic period. This setting is also consistent with prior entropy-based financial studies that compare the pre-pandemic year 2019 with the pandemic year 2020 [37,55]. For the Russia–Ukraine conflict, 24 February 2022 is used as the event date because it marks the outbreak of the military conflict and has been widely used in event-study and entropy-based studies on financial markets [50,56,57]. Thus, the period before 24 February 2022 is defined as the pre-Conflict period, and the period from 24 February 2022 to 31 December 2022 is defined as the during-Conflict period.

## 4. Results

### 4.1. Full-Sample TE Network Construction and Causality Analysis

#### 4.1.1. Original-Scale Causality Analysis

**Figure 1:**
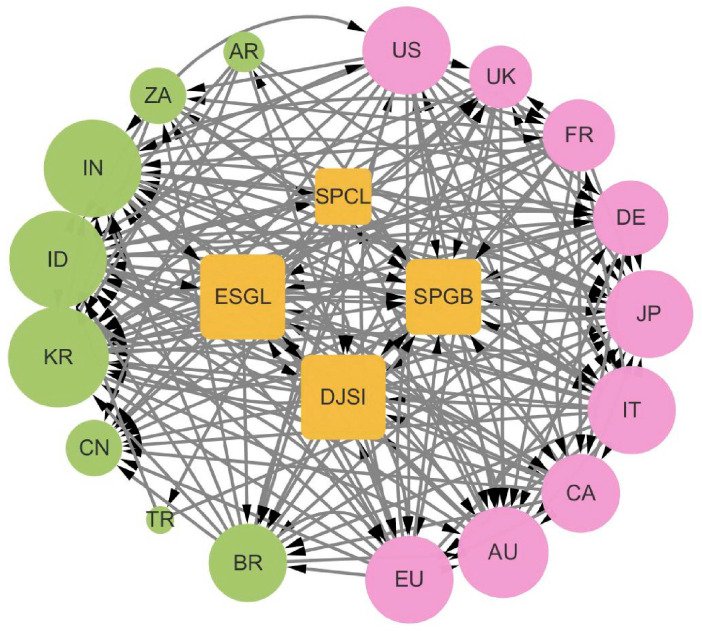
TE causal networks between global banking sectors and green markets. Directed arrows represent direct causal relationships, with arrow direction indicating the direction of causality. Node size reflects the number of direct causal connected links.

In this paper, the information dynamics toolkit IDTxl is used to construct TE causal networks between global banking sectors and green markets. We use the program Cytoscape 3.7.2 for visualization to make the analysis results more comprehensible and visual. Figure 1 delineates the TE causal association structure between the sixteen global banking sectors and four green markets. Purple circles represent banking sector nodes of developed countries, green circles indicate banking sector nodes of emerging countries, and yellow squares stand for green market nodes. These nodes are connected by directed arrows that represent direct causal relations. The direction of the arrow signifies the direction of causality. Each node’s size indicates the number of direct causal connected links it has.

Figure 1 suggests that the structure of the TE causal network between the global banking sectors and the green markets is invariably very complex. There are significant bidirectional causal relationships between the banking sectors and green markets. A massive nonlinear information transmission is directed toward both banking sector nodes and green market nodes. These links reflect two-way interaction channels: banks support green markets through credit provision, underwriting, and portfolio holdings, while shocks in green markets affect banks through asset valuation, funding conditions, and credit risk.

Secondly, the banking sectors of all developed countries play a pivotal role in the TE causal network. This may be attributed to the fact that developed countries have begun the transition to greener and more sustainable economic development at an earlier stage compared to emerging nations. What is more, the banking sectors of developed countries provide greater support in financing and backing green investments and sustainable development innovations [13]. Emerging countries are under pressure to rapidly transition to a green economy, a shift further driven by the need to better balance the environmental, economic, and welfare needs of their citizens [63]. Among emerging countries, three Asian nations—India, Indonesia, and South Korea—exhibit the highest number of causality links. As a representative of emerging nations, South Korea, in collaboration with the banking sector, provided policy loans totaling $30 billion in 2024. By providing incentives for companies to switch to low-carbon production methods, these loans aim to create a more sustainable future.

Lastly, all four green investment indices are closely related to the banking sectors, serving as information transmitters and receivers. This implies that green investments in renewable energy and sustainable development resources also have a spillover effect on the banking sectors of the world’s major economies, in an environment where green finance is becoming a major topic of interest for investors, regulators, and academia. Moreover, different green market indices have varied effects on banking sectors. The S&P Green Bonds Index, Dow Jones Sustainability Index, and MSCI World ESG Leaders Index are three green indices that exhibit higher information spillover to the global banking sectors. By contrast, SPCL is less central, as clean energy equities are more exposed to sector-specific technology and energy-price shocks. These spillovers imply that green finance has financial-stability implications beyond its portfolio-diversification value.

#### 4.1.2. Multiscale Causality Analysis

**Figure 2:**
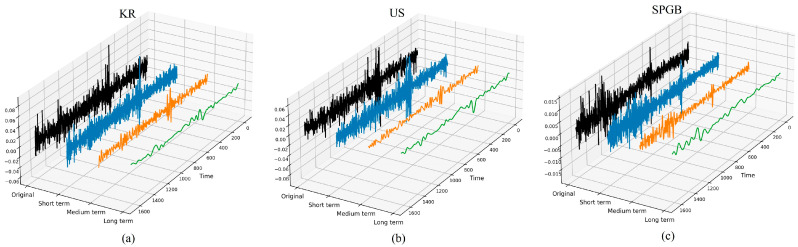
Original and decomposed short-, medium-, and long-term time series for (**a**) KR, (**b**) US, and (**c**) SPGB. The black, blue, orange, and green lines represent the original, short-term, medium-term, and long-term time series, respectively.

We employed the CEEMDAN-SE-KM method for the decomposition and reconstruction of the return time series, as detailed in Section 2.1. Each banking and green market sector time series was reconstructed into three new components, reflecting short-, medium-, and long-term time scales. As an illustration, Figure 2 displays the original and these three scales for the returns of an emerging market (South Korea), a developed market (United States), and a green market (SPGB).

**Figure 3:**
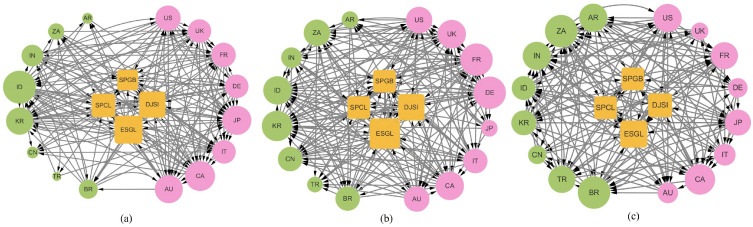
TE causal networks between banking sectors and green markets at three time scales: (**a**) long-term; (**b**) medium-term; (**c**) short-term. Directed arrows represent direct causal relationships, with arrow direction indicating the direction of causality. Node size reflects the number of direct causal connected links.

Figure 2 that shows that both the banking and green markets fluctuate acutely in the short-term scale IMFs, while the medium- and long-term scale IMFs distinctly display cyclical characteristics. Consequently, by decomposing and reconstructing the original yield data, we delineate the TE causality structure between the banking and green investment sectors at different time scales, which can capture the multiscale nature of the causal relations, as illustrated in Figure 3.

**Figure 4:**
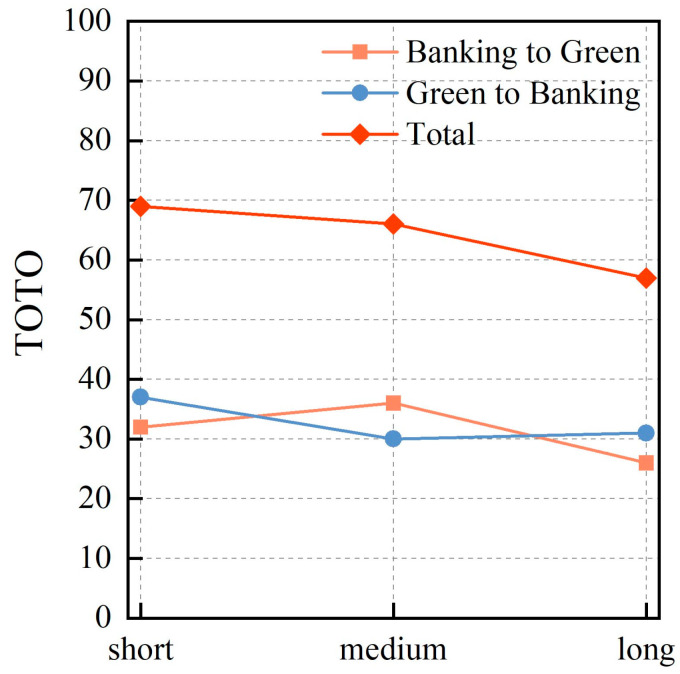
“TOTO” indicator at three time scales.

Figure 3 illustrates the heterogeneity in the causal relationship between banking sectors and green markets across different temporal scales. The short-term network contains the largest number of causal links, with green-to-bank spillovers being strongest at this scale. This pattern is consistent with high-frequency trading, liquidity shocks, sentiment changes, and short-term portfolio rebalancing, through which information from green markets is rapidly transmitted to banking sectors. Compared to the short-term, the significance of emerging countries in the TE causal networks increases in the medium-term and long-term, indicating that the risk spillover between the banking and green sectors in emerging countries is more pronounced over longer time horizons than over short-term cycles. Because the green transition of the emerging economies is itself a long-horizon and financing-intensive process, the linkage between their banking sectors and green markets strengthens as the time scale lengthens. Therefore, when investors are devising risk management strategies, the structure of the TE network at different time scales can provide valuable information for heterogeneous investors with varying investment time scales [31]. In order to gauge the degree of interconnectedness of each sector in the TE causal network, the “TOTO” index is calculated according to Equation (12), as shown in Figure 4.

It is evident from Figure 4 that the total number of transfer entropy causal links is highest at the short-term scale as calculated by the “TOTO” index. This can be interpreted as indicating that information transmission is particularly active in the short-term, with market volatility tending to subside as the time horizon extends [35]. Moreover, at the medium-term scale, the number of causal links from the banking sectors to the green markets is the highest; at the short-term scale, the number of causal links from the green markets to the banking sectors is the greatest. This could be attributed to the fact that traders, speculators, and arbitrageurs are more focused on short-term investments, with currently popular green investments being one of the significant targets for investors seeking short-term gains [24,64]. These findings imply that risk management strategies should be tailored to specific investment horizons: short-term investors should concentrate on sentiment-driven co-movements, while long-term investors and regulators should prioritize attention on persistent transition-related linkages.

### 4.2. Sub-Sample TE Network Construction and Causality Analysis

**Figure 5:**
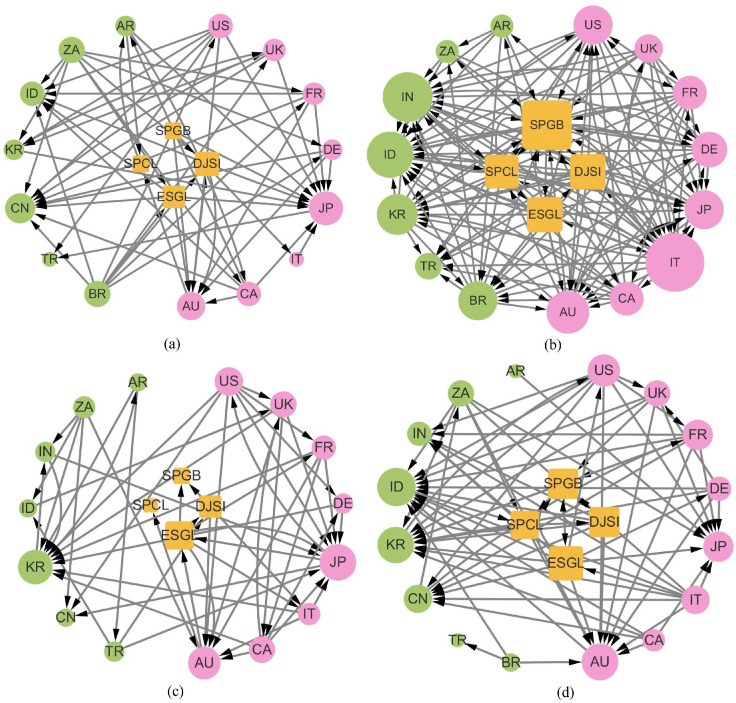
TE causal networks between banking sectors and green markets in crisis events: (**a**) pre-COVID-19; (**b**) during-COVID-19; (**c**) pre-Conflict; (**d**) during-Conflict. Directed arrows represent direct causal relationships, with arrow direction indicating the direction of causality. Node size reflects the number of direct causal connected links.

In this section, we construct TE causal networks between the global banking sectors and the green markets before and after the onset of the COVID-19 pandemic and the Russia–Ukraine conflict. These figures are utilized to analyze the impact of these two crisis events on the causal associations between the banking sectors and the green markets, as depicted in Figure 5.

Figure 5 makes it clear that the TE causal network between the banking sectors and green markets has become more complex since the outbreak of the COVID-19 pandemic and the Russia–Ukraine conflict. This might be a result of increased attention to green investments and sustainable indices by fund managers and investors following the crises. These investments have become more popular because they do not suffer a loss in risk-adjusted returns that are comparable to traditional stocks [65].

Secondly, following the outbreak of the COVID-19 pandemic, the influence of the banking sector in developed countries within the TE causal network has significantly increased. This may be attributed to differences in policy shifts and responses to the COVID-19 pandemic between developed and emerging countries. Based on Equations (12) and (13), we computed “TOTO” and network density, two topological metrics of the TE causal network, before and after the onset of the two crisis events. The results are presented in Table 2.

The “TOTO” indicator, as delineated in Table 2, reveals that subsequent to the emergence of both crises, there was an augmentation in causal relationships from the banking sectors to the green markets and vice versa. Additionally, the network density of the transfer entropy (TE) causal network exhibited an increase following the onset of the COVID-19 pandemic and the Russia–Ukraine conflict, compared to the preceding period. This finding implies an intensification in information transmission between the two markets during the crisis periods. Prior studies utilizing entropy-based methodologies have demonstrated that the COVID-19 pandemic heightened stock market volatility and altered market randomness [55], that the Russia–Ukraine conflict influenced the entropy and volatility of financial time series [56], and that extreme events can manifest distinct signatures in entropy measures by amplifying uncertainty and volatility [57]. Consequently, the increased density of the TE network observed in Table 2 may be indicative of both enhanced cross-market information transmission and a crisis-induced escalation in common risk factors.

In addition, compared to the Russia–Ukraine conflict, the increase in information transmission between the banking sectors and the green markets following the outbreak of the COVID-19 pandemic is more pronounced. This disparity can be attributed to the distinct characteristics of the two crises. The COVID-19 pandemic, as a global public health emergency, has prompted an increased demand from investors worldwide to transition toward a more sustainable and environmentally friendly economy [66]. The pandemic primarily impacted these markets through global uncertainty, liquidity pressures, and a broad demand shock, resulting in a substantial rise in cross-market information flows. The Russia–Ukraine conflict, as a war crisis event, has induced a heightened sense of insecurity among investors. The Russia–Ukraine conflict transmitted shocks more through energy prices, inflation, and geopolitical risk, which affected green assets and then fed into bank credit conditions, thereby diminishing the extent to which their focus shifts toward green investments compared to the impact of the COVID-19 pandemic.

### 4.3. Robustness

**Figure A1:**
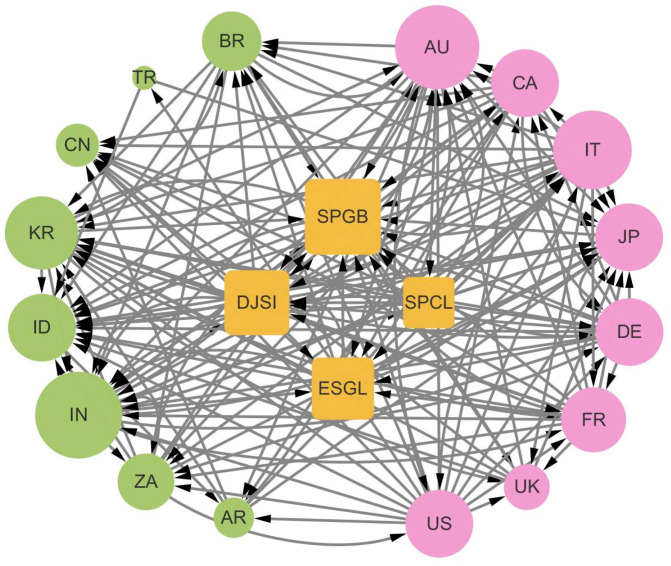
TE causal networks between global banking sectors and green markets with maximum lag (lag = 5). Directed arrows represent direct causal relationships, with arrow direction indicating the direction of causality. Node size reflects the number of direct causal connected links.

To further check the robustness of the results, we add three empirical tests. First, we change the maximum lag in the transfer entropy (TE) estimation from lag = 3 to lag = 5. As shown in Figure A1, the causal network obtained under lag = 5 is close to the benchmark network. The main network structure and the major information-flow pattern remain unchanged. This result suggests that the conclusions are not driven by the selected lag length.

**Figure A2:**
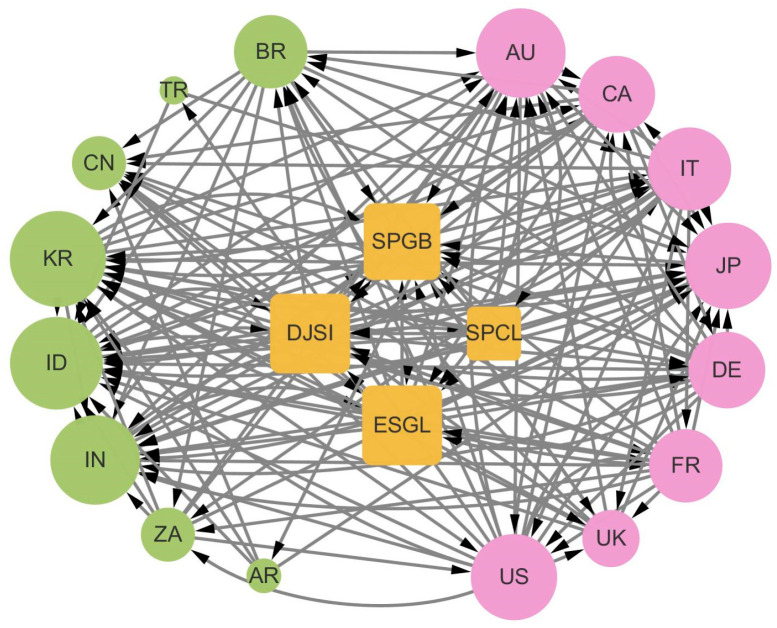
TE causal networks between global banking sectors and green markets (randomly shuffled, *n* = 500 times). Directed arrows represent direct causal relationships, with arrow direction indicating the direction of causality. Node size reflects the number of direct causal connected links.

Second, we increase the number of random shuffling iterations in the significance test from *n* = 200 to *n* = 500. The network shown in Figure A2 is similar to the benchmark result. The major causal links and the overall network pattern are retained. Therefore, the main findings are not sensitive to the number of random shuffling iterations.

**Figure A3:**
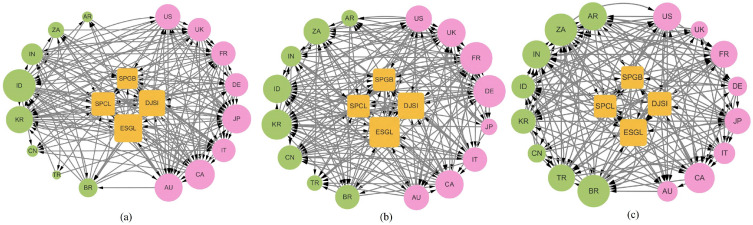
TE causal networks between banking sectors and green markets at three time scales based on the VMD method: (**a**) long-term; (**b**) medium-term; (**c**) short-term. Directed arrows represent direct causal relationships, with arrow direction indicating the direction of causality. Node size reflects the number of direct causal connected links.

Third, and most importantly, we replace the original frequency-domain decomposition procedure with variational mode decomposition (VMD) and rebuild the short-, medium-, and long-term TE causal networks. VMD decomposes a non-stationary series into several modes with different center frequencies by solving a variational problem [67]. The results are reported in Figure A3. The high-frequency and medium-frequency networks are clearly denser than the low-frequency network, which is consistent with the benchmark results. This shows that information transmission between global banking sectors and green markets is stronger at the short- and medium-term scales than at the long-term scale.

Overall, these robustness checks support the main conclusions of this study. The key results can be summarized as follows: (i) the network structures under lag = 5 and *n* = 500 are similar to the benchmark network; (ii) green markets and the banking sectors of developed countries, India, Indonesia, and South Korea remain important nodes; (iii) the high-frequency and medium-frequency networks contain more causal links than the low-frequency network; and (iv) the main direction and pattern of cross-market information flow remain stable.

## 5. Conclusions

The promotion of green economic growth has become a global collective goal for countries in their pursuit of sustainable development. Governments accounting for about 70 percent of global GDP have pledged to achieve net-zero carbon emissions by 2050. The green market has demonstrated an annual growth rate surpassing 100 percent in recent years, with projections indicating that it will account for one-third of global assets by 2025 [24]. The global banking sectors play a significant role in the development of green investments and the fostering of green innovations.

This paper presents the first attempt to apply the transfer entropy method to explore the complex nonlinear and asymmetric causal relations between the global banking sectors and the green markets. We also employ the CEEMDAN-SE-KM approach to investigate the multiscale characteristics of the causal relationship between the two markets. Moreover, this study examines the changes in the associations between the banking sectors and the green markets before and after the outbreak of the COVID-19 pandemic and the Russia–Ukraine Conflict. The following conclusions can be drawn.

Firstly, there are significant bidirectional causal relations between the global banking sectors and the green markets. A massive nonlinear information transmission exists between the two markets. Secondly, the banking sectors in all developed countries play an important part; among emerging countries, three Asian nations—India, Indonesia, and South Korea—have a much more important role. All four of the green investment indices are closely related to the banking sectors. The SPGB, DJSI, and ESGL exhibit higher information spillover to the global banking sectors. Secondly, the causality between the banking sectors and green markets are heterogeneous at different time scales. At the short-term scale, the causality is the most pronounced; the banking sector exerts the greatest influence on the green market at the medium-term scale; and the green market has the most significant impact on the banking sector at the short-term scale. Finally, following the outbreak of the COVID-19 pandemic and the Russia–Ukraine conflict, both crises have impacted the global banking sector and the green market, leading to an increase in the causality between the two markets and a higher efficiency in information transmission. Compared to the Russia–Ukraine conflict, the COVID-19 pandemic has resulted in a more significant increase in causal associations between the global banking sectors and the green markets. The increase in causal linkages is not only driven by stronger underlying economic fundamentals but also reflects heightened market volatility, increased uncertainty, and common external shocks.

These findings are beneficial for regulatory authorities and policymakers in formulating green market policies and dynamic regulation. They also aid in risk management within the volatile financial market environment during crisis events. For green market investors, these discoveries are appealing as they clearly elucidate the information transfer between the green markets and the global banking sectors. This could help investors construct diversified international investment strategies at multiple time scales.

Although this study provides useful evidence on nonlinear information flows between global banking sectors and green markets, several limitations remain. First, the sample covers banking sectors from 16 countries and four green market indices. While these markets are representative, they cannot fully capture global market conditions. Future studies could include more countries, regional markets, and green assets to test the robustness of the results. Second, this study focuses on whether information is transmitted between the two markets, but it does not fully identify the drivers of such transmission. Future research could examine the roles of monetary policy, climate policy, investor attention, energy prices, bank risk, and trade uncertainty. Finally, the results are based on the transfer entropy framework and the CEEMDAN-SE-KM setting, future research could compare transfer entropy with other causality and connectedness methods and test dynamic networks.

## Figures and Tables

**Table 1:** Descriptive statistics of the daily returns.

Code	Mean	Std. Dev.	Min	Max	Skew.	Kurt.	Jarque–Bera	ADF
Panel A: Developed banking sectors								
US	0.0001	0.0129	−0.0990	0.0702	−0.522	10.00	3374.5 ***	−9.01 ***
UK	−0.0002	0.0124	−0.0655	0.0702	−0.194	7.00	1087.4 ***	−7.32 ***
FR	−0.0001	0.0158	−0.0932	0.1100	−0.095	8.71	2197.0 ***	−7.11 ***
DE	−0.0001	0.0172	−0.0970	0.0860	−0.385	6.52	872.2 ***	−8.71 ***
JP	0.0001	0.0102	−0.0687	0.0536	−0.141	7.32	1260.4 ***	−9.24 ***
IT	0.0002	0.0144	−0.0845	0.0732	−0.749	7.65	1604.5 ***	−7.61 ***
CA	0.0001	0.0098	−0.0939	0.1057	−0.350	23.04	27,087.5 ***	−8.51 ***
AU	0.0000	0.0111	−0.0956	0.0782	−0.593	13.77	7899.6 ***	−7.81 ***
Panel B: Emerging banking sectors								
AR	−0.0006	0.0302	−0.3803	0.0942	−3.883	48.29	142,251.5 ***	−8.42 ***
BR	−0.0002	0.0174	−0.1218	0.1010	0.051	6.51	827.1 ***	−7.79 ***
TR	−0.0001	0.0220	−0.1847	0.1112	−0.678	9.86	3289.9 ***	−7.47 ***
CN	−0.0001	0.0085	−0.0357	0.0576	0.157	5.92	578.7 ***	−8.78 ***
KR	−0.0002	0.0130	−0.0700	0.0938	0.267	7.27	1246.8 ***	−7.64 ***
ID	0.0003	0.0118	−0.0790	0.1245	0.117	17.98	15,106.6 ***	−7.34 ***
IN	0.0002	0.0115	−0.0869	0.0787	−0.440	11.51	4922.7 ***	−7.37 ***
ZA	−0.0003	0.0174	−0.1541	0.0843	−0.635	9.50	2949.2 ***	−7.96 ***
Panel C: Green markets								
SPGB	−0.0001	0.0031	−0.0200	0.0151	−0.331	8.81	2302.3 ***	−7.06 ***
DJSI	0.0003	0.0069	−0.0678	0.0497	−0.892	12.95	6884.9 ***	−9.19 ***
SPCL	0.0002	0.0127	−0.0884	0.0784	−0.141	7.73	1508.9 ***	−7.66 ***
ESGL	0.0003	0.0074	−0.0714	0.0552	−0.783	12.57	6336.2 ***	−9.04 ***

Notes: Std. Dev. is the standard deviation; Skew. and Kurt. are the skewness and kurtosis (kurtosis equals 3 under normality); Jarque–Bera is the test statistic for normality; ADF is the augmented Dickey–Fuller unit-root test statistic. *** indicates significance at the 1% level.

**Table 2:** TE causal network topology characteristics in different crisis periods.

Crisis Periods	Total-Out-To-Other		Network Density
	Banking Sectors ⟶ Green Markets	Banking Sectors ← Green Markets	
pre-COVID-19	7	12	0.345
during-COVID-19	21	19	0.8421
pre-Conflict	4	7	0.3157
during-Conflict	8	19	0.4052

## Data Availability

Data on equity and green markets are collected from Thomson Reuters DataStream (https://www.lseg.com/en/data-analytics/datastream-and-macroeconomics), accessed on 14 July 2026.
